# A qualitative study on factors that influence women’s choice of delivery in health facilities in Addis Ababa, Ethiopia

**DOI:** 10.1186/s12884-016-1105-7

**Published:** 2016-10-12

**Authors:** Tigest Shifraw, Yemane Berhane, Hanna Gulema, Tamil Kendall, Anne Austin

**Affiliations:** 1 Addis Continental Institute of Public Health, P.O Box 26571/1000, Addis Ababa, Ethiopia; 2 Maternal Health Task Force, Harvard TH Chan School of Public Health, 651 Huntington Avenue, Boston, MA 02115 USA

**Keywords:** Safe delivery, Institutional delivery, Maternal mortality, Pull factors, Birth preparedness, Urban

## Abstract

**Background:**

Facility based delivery for mothers is one of the proven interventions to reduce maternal and neonatal morbidity and mortality. This study identified women’s reasons for seeking to give birth in a health facility and captured their perceptions of the quality of care they received during their most recent birth, in a population with high utilization of facility based deliveries.

**Methods:**

This qualitative study was conducted in eight health centers in Addis Ababa. Women bringing their index child for first vaccinations were invited to participate in an in-depth interview about their last delivery. Sixteen in-depth interviews were conducted. Interviews were conducted by trained researchers using a semi-structured interview guide. The data were transcribed verbatim in Amharic and translated into English. A thematic analysis was conducted to answer specific study questions.

**Results:**

All research participants expressed a preference for facility based delivery because of their awareness of obstetric complications, and related perceptions that facility-birth is safer for the mother and child. Dimensions of quality of care and the cost of services were identified as influencing decisions about whether to seek care in the public or private sector. Media campaigns, information from social networks and women’s experiences with healthcare providers and facilities influenced care-seeking decisions.

**Conclusions:**

The universal preference for facility-based birth by women in this study indicates that, in Addis Ababa, facility based delivery has become a preferred norm. Sources of information for decision-making and the dimensions of quality prioritized by women should be taken into account to develop interventions to promote facility-based births in other settings.

## Background

Facility-based delivery is one of the proven interventions to reduce maternal and neonatal morbidity and mortality [1]. In recent years the government of Ethiopia has established different approaches to increase facility based births, and reduce maternal mortality. Primary among them has been the expansion of health facilities that offer basic delivery services (vaginal birth for low risk women) to improve the proximity of maternal services, and instituting measures to ensure all maternal services (including obstetrical delivery, and where available, neonatal intensive care) in the public sector are free. While the geographic distribution of these facilities is uneven, it is estimated that almost all residents of Addis Ababa live within a five kilometer radius of a health facility that provides delivery services [2–4]. Urban Ethiopian women are significantly more likely than rural women to deliver in a health facility (54 % versus 4 %), and women living in Addis Ababa have the highest national percentage of institutional delivery, which was 82 % in 2011 [4, 5]. As interventions to reduce the geographic and financial barriers to facility based maternal care are implemented across the country, it is important to understand the factors that have facilitated women’s decision to give birth in health facilities (hospitals or health centers). There is substantial evidence on why Ethiopian women do not deliver in facilities, including evidence that home based deliveries are considered normal, distance to health facilities is a barrier, and a lack of decision making power among pregnant women [6]. A number of studies have identified social and demographic factors that are associated with increased facility-based delivery in Ethiopia. Being an urban resident, primipara status, antenatal care (ANC) attendance during the last pregnancy, women and their husband’s having secondary education or more and women’s knowledge about pregnancy and delivery services have been associated with facility-based delivery service utilization [5–9]. This study identified women’s reasons for seeking a facility-based birth in a population that tends to give birth in hospitals or health centers, and captured their perceptions of the quality of care they received during their most recent birth. Increased understanding of the reasons women in Addis Ababa choose to deliver in facilities, how they select these facilities and their experiences of care can contribute to developing strategies to improve the uptake of facility-based maternity services. Thus, these findings may contribute to the development of interventions to promote increased uptake of facility-based maternal services in other regions of the country, and corresponding declines in maternal mortality and morbidity.

## Methods

### Study setting

This study was conducted in public health facilities that provide childhood immunization services. The government health centers that were sampled in this study, are part of the St. Paul’s Millennium College Hospital referral network, in Addis Ababa, Ethiopia. According to EDHS 2011, the total fertility rate was 1.5 children per woman in Addis Ababa [4].

### Study design, participants and sampling procedure

A qualitative study was undertaken to understand the factors that have contributed to a woman’s choice to deliver in a health facility and her perception of the quality of care provided.

In 2009/10, the Ethiopian government started providing free antenatal care, delivery and postnatal in all public health centers. In Ethiopia, the first vaccination is normally scheduled at six weeks after birth. Postnatal women attending the first routine vaccination for their last born child (index child) in the study period from each of the participating urban health facilities in Addis Ababa were invited to participate. A purposive sampling procedure was used to identify women who were first time mothers as well as women who had had more than one birth from each health facility participating in the study.

Sample size was determined using the principle of “saturation”—women were asked to participate in interviews until additional interviews did not provide additional evidence about the main themes of interest [10].

In-depth interviews were conducted to gather information on personal experiences with seeking facility based care. Interviews were conducted by five researchers with graduate level training in qualitative methods. Informed consent was obtained from all respondents. Interviews were conducted using a semi-structured interview guide, which was prepared in Amharic (local language). On average interviews took about 30 min. Each woman completed only one interview, but interviews were conducted during two time periods (August and December 2013). This allowed data collection and analysis to inform each other mutually [11]. Analysis of the first round of data collection allowed for identification of themes and gaps in the information which were explored in greater depth during interviews conducted during the second period of data collection.

### Data analysis

The tape recorded interviews were transcribed in Amharic and translated into English by the researchers. The translated data were exported into Open Code software to facilitate coding and analysis. Each translated document was coded line-by-line to flag ideas and statements related to the study objective, and then codes were grouped to identify themes that were related to the factors that influence women’s decision to seek institutional delivery. A priori themes were coded based on the study objectives and emergent themes were identified based on the narratives of research participants.

### Ethical considerations

Addis Continental Institute of Public Health ethical review board approved the study. Written informed consent was sought from each participant before the interview began, and after explaining the purpose of the study. The decision to participate in the study was not linked to the service participants were entitled to obtain. Written consent was archived at the Addis Continental Institute of Public Health data management unit. All participants received a unique identification number that was used on the recorded interviews, on the transcripts and also during publication. The entire interview was recorded, and after transcription, the cassette was discarded. Access to the raw data was limited only the research team members. To ensure privacy, all interviews were conducted in a private room within the health facility.

## Results

Eighteen women were invited to participate and 16 accepted. Two women refused to participate due to their busy schedule. Fifteen out of sixteen of the participants were married. The mean age of the participants was 27 years with a standard deviation (SD) of 4.08 years. The mean parity of the participants were 2.06 with a SD of 1.39. Four women delivered via C-section; the other twelve had vaginal births. Ten women delivered in public facilities (health center or hospital), five delivered in private facilities and one delivered at home (Table 1).

**Table 1:** Demographic Characteristics of the respondents (*n* = 16) in selected public health facilities, Addis Ababa, Ethiopia

Variable		Frequency	Percentage
Age Mean = 27 SD = 4.08	18–24	7	43.8
	25–34	8	50.0
	> = 35	1	6.3
Marital status	Married	15	93.8
	Single	1	6.3
Mode of delivery	C-section	4	25.0
	Vaginal delivery	12	75.0
Number of pregnancy	1–3	13	81.3
	4–6	2	12.5
	> = 7	1	6.3
Parity Mean = 2.06 SD = 1.39	1–3	14	87.5
	4–6	2	12.5
Place of delivery	Public facility	10	62.5
	Private facility	5	31.2
	Home	1	6.3

### Awareness of obstetric complications and attitudes towards facility based births

All sixteen women stated that facility-based care is best for mother and child and indicated that the knowledge of potential complications shaped their decision to seek facility based delivery care. Participants had obtained information about potential maternal/newborn complications through the media, through health education provided to them during ANC visits, and other contacts with the health system.

One woman asserted, “*Health center (deliveries) are good because the providers know how to manage the delivery of the baby and placenta. The pregnancy may not be normal - the placenta may come before the baby, or the fetal position may not be correct. This is the reason I delivered at a health center… I heard advertisements from the radio and TV that we should have delivery and follow up care at the health center.” (Participant # 16, Para 1)*

Noting the advantages of receiving birth support from a skilled attendant, one woman remarked*, “I delivered at the health center believing it was better for me and my child. When you give birth at home, so many things can happen…the child may be distressed- so if it (delivery) is here (health facility) the doctor may help you- there is no such help at home”. (Participant # 13, Para 2)*

The one woman who gave birth at home, clearly stated that she would have “*preferred to deliver at a health facility”* and that *“it was an accident*” for her to deliver at home. (Participant # 15, Para2) She also stressed her intention to seek facility-based care for any future deliveries.

### Where to give birth: balancing fears of disrespect and abuse against cost

Women who sought care in public facilities in our sample confirmed that they did not have to pay any user fees. Women’s experiences indicated that this situation had changed over time. For instance, *“Previously, when I delivered (my first) two children I paid fifty-two birr. Now there is no payment. There is even a paper that says no payment.” (Participant # 1, Para 3)*

Payment and perceived quality of care were relevant for women when choosing the health center at which to deliver. The majority of women in this sample had initially sought care in the public sector. Fear of complications and women’s perception that facility based care was the safest mode of delivery trumped rumors of poor quality care in the public sector.

As one woman stated, *“Giving birth at home is not a choice at all. I or my baby might get in trouble. I had heard that (government health facilities) are abusive and do not give you proper care…. But I never thought I would give birth at home; it did not cross my mind at all.” (Participant # 11, Para 1)*

Other women who agreed that delivering at home was not an option, were motivated to seek care in the private sector out of fear of disrespect and abuse during childbirth. All three of the women who gave birth in the private sector said they did so because they would receive more respectful care. A woman said “*Nowadays, no one would want to give birth at home. These days people know better about the advantages of facility-based delivery. But when they choose health facilities, they take health workers attitudes into consideration. When they think of government health facilities they think there is a lot of insults and abuse. So they prefer private facilities”* (Participant # 1, Para 1)

However, the interviews also provided evidence that women’s perceptions of the quality of care in the public sector may be changing. “*Because service (in the public sector) is given free of payment, women used to perceive that they will not get good care. Currently, that is changing, and women are starting to prefer the public health centers*.” (Participant # 3, Para 1) Another theme that emerged was that there were changing perceptions among women on the superior quality of care available in the private sector. *“My friends delivered at a private hospital. No special things were given at private hospital, except spending your money.” (Participant # 3, Para 1)* Another woman noted, *“Here, at the government health center it will cost you nothing in addition they will give you good care and save your money. In a private facility there is good care too but you need to have the money.”* Women also expressed their belief that private facilities promoted cesarean delivery for financial gain. *“If you go to a private clinic they are in a hurry to operate in order to increase their income….”* One woman chose to seek care in the private sector so that she could have an elective cesarean delivery. While no causal relationships can be drawn from this small qualitative sample, it is notable that three of the five women who delivered in the private sector had cesarean deliveries while only one of the 10 women who delivered in the public sector had a cesarean delivery.

#### Positive and negative perceptions of quality of maternity care acquired through social networks

Women reported making decisions about what facilities to attend based on knowledge obtained through their social networks. One woman said “*People say that they had good service here. It is true! It is good for the rich as well as for the poor*.” (Participant # 10, Para 6) The importance of community opinions about facilities was underlined by a woman who explained “*I don’t think I would have come here when I was pregnant if I hadn’t heard (positive) feedback from others*.” (Participant # 1, Para 3)

Women’s experience with ANC or interactions with other aspects of a health center also helped them decide where to deliver. “*When I came (here) for ANC I never encountered a situation where the health provider was not around or available, and whenever I came, I got a good checkup*.” (Participant # 9, Para 2)

#### Improvements over time in the public sector

Quite a few women observed that, “*now people choose [public] health centers; many believe that the service at the health center has improved*.” (Participant # 7, Para 1) One woman also noted that she had heard about government efforts to improve the quality of care in public facilities. “*I heard on the media that there have been improvements, and I decided to deliver my second baby in the public sector*”. (Participant # 2, Para 2) Some women attended public health centers despite having heard about poor quality, or even abusive care but did not experience this. As one woman noted, “*I had heard people say a lot of things about this place. I heard them say that they hit their patients; they mistreat them. I was really scared to come here… I was really worried, but thanks to God, things were not as expected… they were really nice people, and I didn’t encounter any problems” (*Participant # 9, Para 2)

### Experience of care

Most of the women in this sample expressed satisfaction with the attitudes of providers, and chose facilities in which they had previously experienced good care, or facilities where their friends had said good care was provided. *“Previously I delivered two children in this (public) health center at that time the health providers were good that is why I came again for my third delivery. They gave care for pregnant women, respect people and are disciplined. I told others to come in this facility for delivery because of their services.” (Participant # 1, Para 3)*

Another woman, who had given birth in a public facility, noted that *“The very good thing- I got a good doctor. I mean my baby’s weight was high and it was difficult for me. By help of them, thanks to God, it was ok. They (the providers) were cooperative, and helped me to be strong. I experienced this in my delivery.” (Participant # 2, Para 2)* Another woman shared that the providers, “*reassure you not to be stressed…. When labor pain comes, (if) you make inappropriate shouting and sounds- they did not disgrace you- they encouraged you to be strong*” (Participant # 16, Para 1)

The one woman, who had a cesarean delivery in the public sector hospital noted, *“The health workers were good and they were sympathetic towards me. During the surgery, the doctors helped me to relax. I used to be afraid of surgery: I thought I would die. But here, they were talking to me and comforting me while the others did their job. I was very happy.” (Participant # 7, Para 1)*

While almost all women reported being satisfied with the care they received, one woman who had had good experiences in the facility during her previous two births, found the behaviors of providers unacceptable and abusive on her third (most recent) delivery. “*They mistreated me… this isn’t appropriate. I was thinking to write a comment and insert it in the box. I will do that in the future. They used insulting language.”* She went on to state that there was no privacy during the delivery and that the nurses left the door open so that everyone could “*see you*” and that the nurse “*opened the door and did not close it until she finished talking with other nurses at the door*” (Participant # 1, Para 3)

### Facility cleanliness

One factor, noted by many women, was the cleanliness of the health facility. A woman who had delivered in a public facility said that there had been improvements in cleanliness over time, *“At the time of labor there was cleanliness. For example I was bleeding; he (the provider) took me to the toilet and washed me. He poured water for me. Last year I didn’t see this. I am very happy. I think there are a lot of changes.” (Participant # 2, Para 2)* Another woman also observed improvements in cleanliness over time, *“no blood on the bed and there was new paint on the walls- better than earlier.” (Participant # 1, Para 3)* Another woman noted that “*after you are finished (delivering) they clean and prepare the bed (in the postpartum room) before you get there*”. (Participant # 8, Para 3) Cleanliness was valued by women, as this woman who explained “*I say it’s nice because of the cleanliness of the beds and the delivery room… and the provider themselves were clean*” (Participant # 16, Para 1)

Women in the private sector appreciated the cleanliness of the facilities too, *“It was so clean; they changed our clothing and sheets frequently for us. I liked it so much.” (Participant # 11, Para 1)* Additionally, women greatly appreciated that they did not have to give birth in their own clothing, *“They give you their own pajama and shoe. From admission date to discharge date you did not use your own cloth and shoe.” (Participant # 3, para 1)*

## Discussion

This research suggests that facility-based childbirth is a preferred norm in Addis Ababa, indicating that sociocultural barriers to facility delivery that have been identified as important in other Ethiopian settings can be overcome [5, 7, 9, 12–14]. Overall, women in this study had been convinced, through media campaigns and interactions with the health system, that facility based births were best for both the mother and the child. This study supports findings from other studies that suggest that having access to information through modern media influences women’s knowledge about delivery risks and availability of services [15].

In addition to media reports about complications, women explained that they selected particular facilities because of their own and their family and friends’ experiences [13, 16]. This suggests that in Addis Ababa, social networks are important sources for information for decision-making. Women identified cleanliness and respectful, supportive provider attitudes as important pull factors. These findings complement other research with Ethiopian women that has identified poor patient-provider interactions as a barrier to seeking facility-based delivery [16]. In this study, women’s reports of experiencing disrespect and abuse were rare, with only one woman identifying that she had been abused. This is another encouraging improvement, as the government expands access to affordable and acceptable facility delivery for all women [16, 17]. In other studies, cost has been cited as a clear barrier to women seeking facility based care [16, 18]. The Ethiopian Government’s efforts to ensure free maternal services in public facilities appears to be working; participants who delivered in the public sector health facilities stated that previously they paid some amount of money during ANC, delivery and postnatal care, and now they didn’t pay anything. Women also expressed that this encouraged women to seek care in public facilities. This finding agrees with other research that has associated insurance coverage and fee exemptions with higher facility-based delivery rates [19, 20].

Studies have shown that women report better quality of care in private facilities, but that cost can be a deterrent [15]. It is encouraging that when many women, who participated in this study, compared the quality of care received at public and private institutions they noted that there was no real difference, except cost. However, for other women better perceived quality of intrapersonal care was a motivating factor for women to deliver in private facilities.

### Limitations

Our sample consisted of women and children who survived birth. It has been shown that poor quality of care impacts maternal and child survival [1, 14], so our data may be biased towards higher quality of care. Also our respondents were invited to participate at child vaccination clinics, which may indicate that they are more aware or more compliant with health education campaigns and advice given by healthcare providers than other women, and thus may have more positive perceptions of facility-based birth [12, 21]. Additionally the small number of respondents limits the generalizability of the study findings beyond similar groups of urban women. Nevertheless, the consistency of responses among this small group of urban women who sought facility-based birth with regards to their awareness of birth complications and consequent valuation of facility-based delivery and skilled birth attendants (midwives, nurses and physicians); cost, cleanliness and respectful care as important dimensions of quality; and the fact that women made decisions about facility-based birth with information from media, social networks and their own experiences may be useful insights to inform communication and quality improvement initiatives to increase facility-based birth in other Ethiopian settings.

## Conclusions

In this urban setting, facility-based childbirth has become a preferred norm. Women noted fear of obstetric complications and awareness of free delivery services because of media campaigns as primary motivations for seeking facility-based care. This study also highlighted that information gathered from social networks and women’s own experiences with health facilities and healthcare providers before delivery were important for determining where women sought labor and delivery services. Cleanliness, respectful care from providers and cost were key factors identified by women as influencing their decisions and perceptions of quality of care. This suggests that the Ethiopian Government’s efforts to expand access to services by improving infrastructure and removing financial barriers to facility based care must be complemented by efforts to ensure that the care accessed by women is acceptable and of high quality. The importance of social networks and women’s experiences with healthcare facilities and providers prior to delivery for influencing where they chose to give birth suggests that creating opportunities for women and other community members to appreciate the increasing availability and quality of maternity care by visiting facilities could be effective for promoting facility-births as a preferred norm in other areas of Ethiopia.

## Abbreviations

ANC: Antenatal care
FMOH: Federal Ministry of Health
AARHB: Adds Ababa Regional Health Bureau
MHTF: Maternal Health Task Force
